# Quadratic Euler characteristic of symmetric powers of curves

**DOI:** 10.1007/s00229-025-01623-0

**Published:** 2025-03-20

**Authors:** Lukas F. Bröring, Anna M. Viergever

**Affiliations:** 1 Fakultät für Mathematik, Universität Duisburg-Essen, Thea-Leymann-Str. 9, 45127 Essen, Germany; 2 Fakultät für Mathematik, Leibniz Universität Hannover, Welfengarten 1, 30167 Hannover, Germany

**Keywords:** 14G27, 14N10, 14F42

## Abstract

We compute the quadratic Euler characteristic of the symmetric powers of a smooth, projective curve over any field *k* that is not of characteristic two, using the Motivic Gauss–Bonnet Theorem of Levine–Raksit. As an application, we show that the power structure on the Grothendieck–Witt ring introduced by Pajwani–Pál computes the compactly supported ${\mathbb {A}}^1$-Euler characteristic of symmetric powers for all curves.

## Introduction

One can assign a *quadratic Euler characteristic* to a smooth, projective scheme over a field *k* that is not of characteristic 2 using motivic homotopy theory, introduced by Morel–Voevodsky. Quadratic Euler characteristics were first introduced by Hoyois [13] and are elements of the Grothendieck–Witt ring ${{\,\textrm{GW}\,}}(k)$ of quadratic forms over *k*. These quadratic forms carry a lot of information: If $k\subset {\mathbb {R}}$, Levine [16, Remark 2.3(1) and Proposition 2.4(6)] proved that the signature of the quadratic Euler characteristic $\chi (X/k)$ of a smooth projective scheme *X* over *k* is equal to the topological Euler characteristic of $X({\mathbb {R}})$ and the rank of $\chi (X/k)$ is the topological Euler characteristic of $X({\mathbb {C}})$. In practice however, $\chi (X/k)$ is generally hard to compute. Quadratic Euler characteristics are often used in the program of refined enumerative geometry, which aims to obtain “quadratically enriched” versions of results in classical enumerative geometry.

Our main theorem is the following.

### Theorem 1

Let *C* be a smooth, projective curve of genus *g* over a field *k* that is not of characteristic 2. Let $n\in {\mathbb {Z}}_{\ge 1}$. Then if $n = 2m$ is even, we have

$$\begin{aligned} \chi ({{\,\textrm{Sym}\,}}^{n}C/k)&= \sum _{i=0}^m\left( {\begin{array}{c}g\\ i\end{array}}\right) \langle -1\rangle ^i + \frac{1}{2}\left( \left( {\begin{array}{c}2g-2\\ n\end{array}}\right) - \sum _{i=0}^m\left( {\begin{array}{c}g\\ i\end{array}}\right) \right) H\in {{\,\textrm{GW}\,}}(k)\\&= \frac{1}{2}\left( \left( {\begin{array}{c}2g-2\\ n\end{array}}\right) + (-1)^m\left( {\begin{array}{c}g-1\\ m\end{array}}\right) \right) \cdot \langle 1\rangle \\&\quad + \frac{1}{2} \left( \left( {\begin{array}{c}2g-2\\ n\end{array}}\right) -(-1)^m\left( {\begin{array}{c}g-1\\ m\end{array}}\right) \right) \cdot \langle -1\rangle \end{aligned}$$

and if *n* is odd, we have

$$\begin{aligned} \chi ({{\,\textrm{Sym}\,}}^{n}C/k) = -\frac{1}{2} \left( {\begin{array}{c}2g-2\\ n\end{array}}\right) H\in {{\,\textrm{GW}\,}}(k), \end{aligned}$$

where $\langle a\rangle \in {{\,\textrm{GW}\,}}(k)$ for $a \in k^\times$ denotes the quadratic form $x \mapsto ax^2$ and $H = \langle 1\rangle + \langle -1\rangle \in {{\,\textrm{GW}\,}}(k)$ is the hyperbolic form.

### Remark 2

We note that if $n=1$, Theorem 1 states that $\chi (C/k) = (1-g)H$. This was already known: given that the dimension of *C* is odd, it follows from the Motivic Gauss–Bonnet Theorem proven by Levine and Raksit [18, Corollary 8.7] that $\chi (C/k) = m\cdot H$, where

$$\begin{aligned} m = \dim _kH^0(C, \mathcal {O}_C) - \dim _kH^0(C,\Omega _C) = 1-g. \end{aligned}$$

Here, $\Omega _C$ denotes the sheaf of Kähler differentials of *C*.

### Remark 3

For a smooth, projective curve *C*, the schemes ${{\,\textrm{Sym}\,}}^nC$ are again smooth and projective, see Proposition 11. Thus, the quadratic Euler characteristic of ${{\,\textrm{Sym}\,}}^nC$ is well defined.

### Remark 4

MacDonald [19, (4.4)] computed the topological Euler characteristic of ${{\,\textrm{Sym}\,}}^nC$ for a smooth, projective complex curve *C* to be

$$\begin{aligned} \chi ^{top}({{\,\textrm{Sym}\,}}^nC) = (-1)^n\left( {\begin{array}{c}2g-2\\ n\end{array}}\right) . \end{aligned}$$

This implies that if *C* is a smooth, projective curve over a field *k* of characteristic zero, we have

$$\begin{aligned} \operatorname {rank}(\chi ({{\,\textrm{Sym}\,}}^nC/k)) = (-1)^n\left( {\begin{array}{c}2g-2\\ n\end{array}}\right) , \end{aligned}$$

which matches with the rank of the forms in Theorem 1. Indeed, there exists a smooth, projective curve $C_0$ over a subfield $k_0 \subset k$ with the following two properties: First, $k_0$ is finitely generated over $\mathbb {Q}$ and, second, the base change $(C_0)_k$ to *k* is isomorphic to *C*. Since the rank of the quadratic Euler characteristic is invariant under base-change and $k_0$ admits an embedding into $\mathbb {C}$, we get after choosing an embedding $k_0 \rightarrow \mathbb {C}$ that

$$\begin{aligned} \operatorname {rank}(\chi ({{\,\textrm{Sym}\,}}^nC/k))&= \operatorname {rank}(\chi ({{\,\textrm{Sym}\,}}^nC_0/k_0))\\&=\operatorname {rank}(\chi ({{\,\textrm{Sym}\,}}^n(C_0)_\mathbb {C}/\mathbb {C}))\\&= (-1)^n\left( {\begin{array}{c}2g-2\\ n\end{array}}\right) \end{aligned}$$

as desired.

### Remark 5

The formula in Theorem 1 evaluates to zero for a smooth, projective curve *C* of genus *g* whenever $n > 2g-2$. If *C* has a rational point, this can also be seen with the following brief argument: Consider the map ${{\,\textrm{Sym}\,}}^nC\rightarrow \text {Pic}^n(C)$ sending a point on ${{\,\textrm{Sym}\,}}^nC$ with associated divisor *D* to $\mathcal {O}_C(D)$. By for example the proof of [24, Theorem 7.33], this map realizes ${{\,\textrm{Sym}\,}}^nC$ as a Zariski locally trivial projective space bundle over $\text {Pic}^n(C)$ and using the rational point we can find an isomorphism $\text {Pic}^n(C)\cong \text {Pic}^0(C)$. Using the Motivic Gauss–Bonnet Theorem, the quadratic Euler characteristic of any Abelian variety is zero because its tangent bundle is trivial. Combining this with [16, Proposition 2.4] yields the desired vanishing.

As an application, we extend a result on compatibility of power structures of Pajwani and Pál [26]: Over a field *k* of characteristic zero, Arcila-Maya et al. [2] extended the quadratic Euler characteristic to a motivic measure

$$\begin{aligned} \chi _c: K_0(\text {Var}_k)\rightarrow {{\,\textrm{GW}\,}}(k) \end{aligned}$$

called the compactly supported ${\mathbb {A}}^1$-Euler characteristic. On $K_0(\text {Var}_k)$, Gusein-Zade et al. [9] showed that symmetric powers of schemes define a power structure. In [26], Pajwani and Pál constructed a power structure $a_*$ on ${{\,\textrm{GW}\,}}(k)$ with the following property: If there exists a power structure on ${{\,\textrm{GW}\,}}(k)$ such that $\chi _c$ respects the power structures, then the power structure on ${{\,\textrm{GW}\,}}(k)$ has to agree with $a_*$. It is still an open question, whether there indeed is a power structure on ${{\,\textrm{GW}\,}}(k)$ such that $\chi _c$ respects the power structures. In Theorem 27, we prove that if ${{\,\textrm{GW}\,}}(k)$ is endowed with the power structure $a_*$, then $\chi _c$ respects the power structures for all curves over a field of characteristic zero.

At the moment, there is no general formula for the quadratic Euler characteristic of a quotient scheme. Theorem 1 is an example result for this and might be helpful for understanding the general case. Further work on quadratic Euler characteristics of quotient schemes will appear in the upcoming PhD thesis of the first named author.

The proof of Theorem 1 uses an explicit computation in conjunction with the Motivic Gauss–Bonnet Theorem proven by Levine and Raksit [18], which computes the quadratic Euler characteristic of a smooth projective scheme as a composition of a cup product and trace on Hodge cohomology.

In related work, the Motivic Gauss–Bonnet Theorem was also used by Levine et al. [17] to compute the quadratic Euler characteristic of a smooth hypersurface in ${\mathbb {P}}^n$ and in the second named author’s computation of the quadratic Euler characteristic of a smooth same-degree complete intersection in ${\mathbb {P}}^n$, see [34].

Adjacent to our application, Pajwani and Pál [26] showed that their power structure is respected for zero dimensional schemes. Work of Pajwani, Rohrbach, and the second named author [27] extended this to the class of “étale linear varieties”, which is the subring of $K_0(\text {Var}_k)$ generated by the class of $[{\mathbb {A}}^1]$ and the classes of the form $[\text {Spec}(L)]$ where *L* is a finite étale algebra over *k*. Since curves are almost always outside of the class of “étale linear varieties” by [27, Corollary 6.6], Theorem 27 yields a proper extension of the class of varieties for which the power structure is respected.

We would like to highlight that Theorem 1 has been proven independently by Simon Pepin Lehalleur and Lenny Taelman, as part of work in progress on the higher dimensional case.

### Notation and conventions

Throughout, we let *k* be a field that is not of characteristic 2. By a curve, we mean a one-dimensional, geometrically connected, separated, finite-type scheme over *k*. For a smooth, projective scheme *X* over *k*, we write $\Omega _X$ for the sheaf of Kähler differentials on *X*. For $i\in {\mathbb {Z}}_{\ge 0}$, we write $\Omega _X^i = \Lambda ^i\Omega _X$.

For $p,q, p', q' \in \mathbb {Z}_{\ge 0}$, we denote the cup product of $a \in H^q(X,\Omega ^p_X)$ and $b \in H^{q'}(X,\Omega ^{p'}_X)$ by $ab:= a\cdot b:= a \cup b \in H^{q+q'}(X,\Omega ^{p+p'}_X)$. This cup-product is graded commutative if we consider $a \in H^q(X,\Omega ^p_X)$ to be of degree $q-p$. That is, we have for $a \in H^q(X,\Omega ^p_X)$ and $b \in H^{q'}(X,\Omega ^{p'}_X)$ that $ab = (-1)^{(q-p)(q'-p')} ba$.

We let $K_0(\text {Var}_k)$ be the Grothendieck group of varieties over *k*. This is the free Abelian group generated by isomorphism classes [*X*] of varieties *X*/*k* modulo the relation that for $Z\subset X$ a closed subvariety we have $[X] = [Z] + [X{\setminus } Z]$. The product rule $[X][Y] = [X\times _kY]$ induces a ring structure on $K_0(\text {Var}_k)$.

For a quasi-compact and quasi-separated scheme *X*, we denote its stable motivic homotopy category as described by Hoyois [14] by $\mathcal{S}\mathcal{H}(X)$. We denote the unit object with respect to the smash product in $\mathcal{S}\mathcal{H}(X)$ by $1_X\in \mathcal{S}\mathcal{H}(X)$. For a ${\mathbb {P}}^1$-ring spectrum $E\in \mathcal{S}\mathcal{H}(X)$ and $Y\in \mathcal{S}\mathcal{H}(X)$, we write $E^{a,b}(Y) = \text {Hom}_{\mathcal{S}\mathcal{H}(X)}(Y, S^{a,b}\wedge E)$ where $S^{a,b}$ denotes the bigraded sphere in $\mathcal{S}\mathcal{H}(X)$ and $\wedge$ denotes the smash product of $\mathcal{S}\mathcal{H}(X)$. We write $\pi ^{a,b}(Y)$ if *E* is the unit.

## Quadratic Euler characteristic

The quadratic Euler characteristic, which was first studied by Hoyois [13], is a refinement of the topological Euler characteristic. We briefly recall its construction.

To *k*, we can associate the stable motivic homotopy category $\mathcal{S}\mathcal{H}(k)$ constructed by Morel–Voevodsky, see [23, 35], which is a symmetric monoidal category. See also Hoyois [14] for an introduction to $\mathcal{S}\mathcal{H}(k)$. Given a smooth projective scheme *X*/*k* with structure map $p_X: X\rightarrow {{\,\textrm{Spec}\,}}(k)$, the object $(p_X)_*1_X\in \mathcal{S}\mathcal{H}(k)$ is strongly dualizable by for example [14, Theorem 5.22]. One can now apply the categorical Euler characteristic of Dold–Puppe [6] to $(p_X)_*1_X$, which yields an endomorphism of the unit of $\mathcal{S}\mathcal{H}(k)$. Morel has proven that ${{\,\textrm{End}\,}}_{\mathcal{S}\mathcal{H}(k)}(1_k)\cong {{\,\textrm{GW}\,}}(k)$, see [22, Theorem 6.4.1 and Remark 6.4.2], and thus we obtain the quadratic Euler characteristic $\chi (X/k)\in {{\,\textrm{GW}\,}}(k)$ as an element of the Grothendieck–Witt ring of quadratic forms over *k*, which we introduce below.

### Definition 6

The *Grothendieck–Witt ring* ${{\,\textrm{GW}\,}}(k)$ over *k* is defined as the group completion of the monoid of isometry classes of non-degenerate quadratic forms over *k* with respect to taking orthogonal direct sums.

The group ${{\,\textrm{GW}\,}}(k)$ inherits a ring structure from the tensor product of quadratic forms.

### Remark 7

Over a field of characteristic not 2, a non-degenerate quadratic form is the same as a non-degenerate symmetric bilinear form, and elementary linear algebra computations yield the following presentation. The quadratic forms $\langle a \rangle :x \mapsto ax^2$ for $x \in k^\times$ generate ${{\,\textrm{GW}\,}}(k)$ and they are subject to the following relations for $a, b\in k^\times$:

- $$\langle a\rangle \cdot \langle b\rangle = \langle a b \rangle $$,
- $$\langle a\rangle + \langle b\rangle = \langle a+b\rangle + \langle ab(a+b)\rangle $$, whenever $$a+ b \in k^\times $$,
- $$\langle ab^2\rangle = \langle a\rangle $$, and
- $$\langle a\rangle + \langle -a\rangle = \langle 1\rangle + \langle -1\rangle =: H$$.

The quadratic form $H = \langle 1\rangle + \langle -1\rangle$ is called the *hyperbolic form*.

Witt [36, Section 1] first described this presentation. In the form presented here, it is [23, Lemma 3.9]. There, the statement is deduced from the statement for Witt rings, proven by Milnor and Husemoller [21, Lemma (1.1) in Chapter 4]. We note that the first relation above does not appear in [23, Lemma 3.9], but we include it here to describe the ring structure of ${{\,\textrm{GW}\,}}(k)$.

See [16, Section 2] for a detailed exposition of the quadratic Euler characteristic and its basic properties.

### Remark 8

In [16, Remark 2.3 (1) and Proposition 2.4 (6)], Levine shows that for $k \subset \mathbb {R}$ and *X* a smooth, projective scheme over *k*, the rank of $\chi (X/k)$ agrees with the topological Euler characteristic of $X({\mathbb {C}})$ and the signature agrees with the topological Euler characteristic of $X(\mathbb {R})$. By a theorem of Saito [29, Theorem 2], the discriminant of $\chi (X/k)$ can also be interpreted in terms of the determinant of $\ell$-adic cohomology for any $\ell$ which is coprime to the characteristic of *k*. See [25, Theorem 2.22] and the surrounding text, for a more detailed discussion.

The Motivic Gauss–Bonnet Theorem of Levine–Raksit [18] provides a way to compute the quadratic Euler characteristic using Hodge cohomology. A more general version has been proved by Déglise–Jin–Khan [5]. We do not state the theorem in full generality here, but rather a simplified version of one of its corollaries, which is enough to prove Theorem 1.

### Theorem 9

(Motivic Gauss–Bonnet, [18, Corollary 8.7]) Let *X* be a smooth, projective scheme over *k*.

- If $\dim X$ is odd, then $\chi (X/k) = m\cdot H$ for some $m \in \mathbb {Z}$.
- If $\dim X = 2n$ is even, then $\chi (X/k) = Q + m\cdot H$ for some $m \in \mathbb {Z}$ where *Q* is the quadratic form given by the composition
  $$\begin{aligned} H^n(X,\Omega ^n_X)\times H^n(X,\Omega ^n_X) \xrightarrow {\cup } H^{2n}(X,\Omega _X^{2n}) \xrightarrow {{{\,\textrm{Tr}\,}}} k. \end{aligned}$$
  Here, $\cup$ denotes the cup product and ${{\,\textrm{Tr}\,}}$ the trace map from Serre duality.

### Remark 10

In [18], the above theorem is stated with the additional assumption that *k* is a perfect field. The statement holds over any field which is not of characteristic 2 by [16, Remark 2.1 (2)]. Namely, let *k* be a field of positive characteristic not 2 and let $k^{\text {perf}}$ be the perfect closure of *k*. Then it is shown in [16, Remark 2.1 (2)] that the extension $k\subset k^{\text {perf}}$ yields an isomorphism ${{\,\textrm{GW}\,}}(k)\cong {{\,\textrm{GW}\,}}(k^{\text {perf}})$. For *X* a smooth projective scheme over *k*, this implies that $\chi (X/k) = \chi (X_{k^{\text {perf}}}/k^{\text {perf}})\in {{\,\textrm{GW}\,}}(k)$, by [16, Proposition 2.4 (6)]. Using flat base-change for cohomology [33, Tag 02KH], we see that the above theorem also holds for non-perfect base fields.

Arcila-Maya et al. [2] constructed a motivic measure $\chi _c:K_0(\text {Var}_k)\rightarrow {{\,\textrm{GW}\,}}(k)$, called the compactly supported $\mathbb {A}^1$-Euler characteristic, which extends the quadratic Euler characteristic of smooth, projective schemes to varieties over *k* when *k* has characteristic 0. Levine et al. [17, Section 5.1] give a definition of $\chi _c$ which works for any field which is not of characteristic two. We briefly recall the construction here.

If *X* is a variety over a field *k* of characteristic zero with structure map $p_X: X\rightarrow \text {Spec}(k)$, one can show using resolution of singularities that the exceptional pushforward $(p_X)_!(1_X)\in \mathcal{S}\mathcal{H}(k)$ is a strongly dualizable object. The categorical Euler characteristic of $(p_X)_!(1_X)$ then computes the compactly supported ${\mathbb {A}}^1$-Euler characteristic $\chi _c(X/k)\in {{\,\textrm{GW}\,}}(k)$.

If *X* is a variety over a field *k* of characteristic $p>0$ with structure map $p_X: X\rightarrow \text {Spec}(k)$, one can show using results of Riou that $(p_X)_!(1_X)\in \mathcal{S}\mathcal{H}(k)$ is a strongly dualizable object after passing to the perfect completion $k^{perf}$ of *k* and inverting *p*. We have that ${{\,\textrm{GW}\,}}(k)\cong {{\,\textrm{GW}\,}}(k^{perf})$ where *k* is the perfect completion of *k*, see [16, Remark 2.1 (2)]. The categorical Euler characteristic of $(p_X)_!(1_X)$ then yields the Euler characteristic $\chi _c(X/k)\in {{\,\textrm{GW}\,}}(k)[\frac{1}{p}]$. One then shows that $\chi _c(X/k)$ lies in the image of the injective map ${{\,\textrm{GW}\,}}(k)\rightarrow {{\,\textrm{GW}\,}}(k)[\frac{1}{p}]$, so that one can define the compactly supported ${\mathbb {A}}^1$-Euler characteristic as an element $\chi _c(X/k)\in {{\,\textrm{GW}\,}}(k)$.

## Symmetric powers of curves

Let *C* be a smooth, projective curve of genus *g* over *k*. For $n\in {\mathbb {Z}}_{\ge 1}$, let ${{\,\textrm{Sym}\,}}^nC = C^n/S_n$ be the quotient of $C^n$ under the action of the symmetric group $S_n$ that permutes the factors. The following results are probably standard, but we include proofs here as we have not been able to find a good reference in the literature.

### Proposition 11

For $n\in {\mathbb {Z}}_{\ge 1}$, the symmetric power ${{\,\textrm{Sym}\,}}^nC$ is smooth.

### Proof

We note that ${{\,\textrm{Sym}\,}}^nC$ is precisely the Hilbert scheme of *n*-points on *C*. By [11, Theorem 1.1(c)], a point $x\in {{\,\textrm{Sym}\,}}^nC$ representing a local complete intersection $X\subset C$ is smooth if $H^1(X, N) = 0$, where *N* is the normal sheaf at *X*. As *C* is a curve, all zero-dimensional subschemes consisting of finitely many points are local complete intersections. Furthermore, $H^1(X, N) = 0$ for all such *X* as they are zero dimensional. Thus, all points are smooth, and so is ${{\,\textrm{Sym}\,}}^nC$. $\square$

The smoothness of ${{\,\textrm{Sym}\,}}^nC$ implies the following result, which we will use in the proof of Theorem 1.

### Proposition 12

Let $p,q\in {\mathbb {Z}}_{\ge 0}$. Assume that *k* has characteristic zero. Then, the quotient map $\pi :C^n\rightarrow {{\,\textrm{Sym}\,}}^nC$ induces an isomorphism

$$\begin{aligned} H^q({{\,\textrm{Sym}\,}}^nC,\Omega ^p_{{{\,\textrm{Sym}\,}}^nC}) \cong (H^q(C^n,\Omega ^p_{C^n}))^{S_n}. \end{aligned}$$

### Proof

The $S_n$-action on $C^n$ induces an $S_n$-action on $\pi _*\Omega ^p_{C^n}$ and $\pi _*\Omega ^p_{C^n}$ is a locally free coherent sheaf since ${{\,\textrm{Sym}\,}}^nC$ is smooth. In particular, since *n*! is invertible in *k*, the sheaf $\pi _*\Omega ^p_{C^n}$ is semistable as an $S_n$-representation and $(\pi _*\Omega ^p_{C^n})^{S_n}$ is a direct summand of $\pi _*\Omega ^p_{C^n}$ and hence also a locally free sheaf on ${{\,\textrm{Sym}\,}}^nC$. Note that taking invariants makes sense because the $S_n$-action on ${{\,\textrm{Sym}\,}}^nC$ is trivial.

Furthermore, the quotient map induces a canonical map $\varphi _C:\Omega ^p_{{{\,\textrm{Sym}\,}}^nC} \rightarrow (\pi _*\Omega ^p_{C^n})^{S_n}$. If $C = \mathbb {A}^1$, then $\varphi _{\mathbb {A}^1}$ is an isomorphism by [7, Theorem 2.2.2] and [4, Chapitre V, §5, Théoréme 4]. For a general curve *C*, we may pass to an algebraic closure to check that $\varphi _C$ is an isomorphism. There, we may represent a closed point $p \in {{\,\textrm{Sym}\,}}^nC$ as the image of $(x_1, \dots , x_n) \in C^n$. Since *C* is a smooth curve, we can find an étale morphism $\psi :U \rightarrow \mathbb {A}^1$ such that $U\subset C$ is an open subscheme containing $x_1, \dots , x_n$. The étale morphism $\psi$ induces an étale morphism ${{\,\textrm{Sym}\,}}^nU \rightarrow {{\,\textrm{Sym}\,}}^n\mathbb {A}^1$. Hence, we get that $\varphi _U$ is an isomorphism from the fact that $\varphi _{\mathbb {A}^1}$ is an isomorphism. Since the inclusion $U\hookrightarrow C$ induces an open immersion ${{\,\textrm{Sym}\,}}^nU \hookrightarrow {{\,\textrm{Sym}\,}}^nC$ and we can cover ${{\,\textrm{Sym}\,}}^nC$ by such subsets, we get that $\varphi _C$ is an isomorphism.

Since $\pi _*\Omega ^p_{C^n}$ is a semistable $S_n$-representation, we obtain a decomposition into isotypical components

$$\begin{aligned} \pi _*\Omega ^p_{C^n} = \bigoplus _\rho (\pi _*\Omega ^p_{C^n})^\rho \end{aligned}$$

where $\rho$ ranges over all irreducible characters.

Now,

$$\begin{aligned} \bigoplus _\rho H^q({{\,\textrm{Sym}\,}}^nC,(\pi _*\Omega ^p_{C^n})^\rho )&= H^q({{\,\textrm{Sym}\,}}^nC,\bigoplus _\rho (\pi _*\Omega ^p_{C^n})^\rho )\\&= H^q({{\,\textrm{Sym}\,}}^nC,\pi _*\Omega ^p_{C^n}) \\&= \bigoplus _\rho H^q({{\,\textrm{Sym}\,}}^nC,\pi _*\Omega ^p_{C^n})^\rho , \end{aligned}$$

where $\bigoplus _\rho H^q({{\,\textrm{Sym}\,}}^nC,\pi _*\Omega ^p_{C^n})^\rho$ is again the decomposition into isotypical components. Since *C* is projective, *C* is separated. Hence, the Čech cohomology of any open affine cover $\mathfrak {U}$ of ${{\,\textrm{Sym}\,}}^nC$ computes sheaf cohomology. The decomposition into isotypical components of $\pi _*\Omega ^p_{C^n}$ induces a decomposition of the Čech complex of $\pi _*\Omega ^p$ into isotypical components with $\check{C}(\mathfrak U, (\pi _*\Omega ^p)^\rho ) = \check{C}(\mathfrak U, \pi _*\Omega ^p)^\rho$ for every character $\rho$. Since taking isotypical components is exact, we hence get $H^q({{\,\textrm{Sym}\,}}^nC, (\pi _*\Omega ^p_{C^n})^\rho ) = H^q({{\,\textrm{Sym}\,}}^nC, \pi _*\Omega ^p_{C^n})^\rho$.

In particular, we have

$$\begin{aligned} H^q({{\,\textrm{Sym}\,}}^nC,\Omega ^p_{{{\,\textrm{Sym}\,}}^nC}) = H^q({{\,\textrm{Sym}\,}}^nC,(\pi _*\Omega ^p_{C^n})^{S_n}) = H^q({{\,\textrm{Sym}\,}}^nC,\pi _*\Omega ^p_{C^n})^{S_n}. \end{aligned}$$

Since $\pi$ is finite, and therefore in particular affine, $\pi ^*$ now induces an $S_n$-equivariant isomorphism $H^q({{\,\textrm{Sym}\,}}^nC,\pi _*\Omega ^p_{C^n})\cong H^q(C^n,\Omega ^p_{C^n})$ by [33, Tag 0G9R] and thus, we get

$$\begin{aligned} H^q({{\,\textrm{Sym}\,}}^nC,\Omega ^p_{{{\,\textrm{Sym}\,}}^nC}) = H^q({{\,\textrm{Sym}\,}}^nC,\pi _*\Omega ^p_{C^n})^{S_n}\cong H^q(C^n,\Omega ^p_{C^n})^{S_n}, \end{aligned}$$

as required. $$\square $$

## Proof of Theorem 1

Let *C* be a smooth, projective curve of genus *g* over *k* and let $n\in {\mathbb {Z}}_{\ge 1}$. In order to prove Theorem 1, we first reduce to the case that *k* has characteristic zero.

Hornbostel [12, Theorem 5.5] constructed a ${\mathbb {P}}^1$-ring spectrum $\textrm{KQ}_S \in \mathcal{S}\mathcal{H}(S)$ for *S* a regular scheme with $2 \in \mathcal {O}_S(S)^\times$ representing Hermitian K-theory. This spectrum satisfies $\textrm{KQ}^{0,0}_k(k) = {{\,\textrm{GW}\,}}(k)$. We show that the unit map of this spectrum is an isomorphism whose inverse induces Morel’s identification of the Grothendieck–Witt ring with the endomorphism ring of the unit of $\mathcal{S}\mathcal{H}(k)$, see [22, Theorem 6.4.1 and Remark 6.4.2]. This answers a question asked by Hornbostel [12, 6.9] and provides a proof for a statement which has long been a folklore theorem in the field. The argument below came out of a discussion with Marc Levine.

### Proposition 13

Write $\textrm{KQ}:= \textrm{KQ}_k$. Let $u:1_k\rightarrow \textrm{KQ}$ be the unit map of Hermitian *K*-theory and also write $u:= \pi ^{0,0}u$ for the induced map on (0, 0)-th stable homotopy groups. Let $M:{{\,\textrm{GW}\,}}(k)\rightarrow \pi ^{0,0}(1_k)$ be Morel’s isomorphism. The composition

$$\begin{aligned} {{\,\textrm{GW}\,}}(k) \xrightarrow {M} \pi ^{0,0}(1_k) \xrightarrow {u} \textrm{KQ}^{0,0}(k) \cong {{\,\textrm{GW}\,}}(k) \end{aligned}$$

is the identity on ${{\,\textrm{GW}\,}}(k)$. In other words, the unit map *u* of Hermitian *K*-theory is an isomorphism and induces the inverse to Morel’s identification morphism.

### Proof

We start by recalling the construction of *M*. Let $a\in k^\times$, then *a* gives rise to the multiplication map

$$\begin{aligned} m_a:{\mathbb {P}}^1\rightarrow {\mathbb {P}}^1, [x:y]\mapsto [ax: y] \end{aligned}$$

on ${\mathbb {P}}^1$. Now *M* maps $\langle a \rangle \in {{\,\textrm{GW}\,}}(k)$ to $m_a$ under the identification $\pi ^{0,0}(1_k)\cong \pi ^{2,1}({\mathbb {P}}^1,\infty )$.

Consider the ${{\,\textrm{SL}\,}}$-oriented vector bundle $p:{\mathbb {A}}^1\rightarrow {{\,\textrm{Spec}\,}}(k)$ with the canonical trivialization of its determinant. Panin and Walter [28, Theorem 5.1] proved that the spectrum $\textrm{KQ}$ is an ${{\,\textrm{SL}\,}}$-oriented cohomology theory. For more details about ${{\,\textrm{SL}\,}}$-oriented cohomology theories, we refer the reader to Ananyevskiy [1] or to [28]. For *X* a smooth scheme over *k*, Panin and Walter also provide an identification of $\textrm{KQ}^{2n,n}(X)$ with the Grothendieck–Witt ring of symmetric objects of degree *n* for the category of bounded chain complexes of vector bundles on *X*; see [28, Sections 4 and 5] for details and precise definitions. This implies that there is a Thom isomorphism

$$\begin{aligned} {{\,\textrm{th}\,}}:\textrm{KQ}^{0,0}(k)\rightarrow \textrm{KQ}^{2,1}({{\,\textrm{Th}\,}}({\mathbb {A}}^1)) \end{aligned}$$

associated with *p*. Here, ${{\,\textrm{Th}\,}}({\mathbb {A}}^1) = {\mathbb {A}}^1/ ({\mathbb {A}}^1{\setminus } \{0\})$ is the Thom space of ${\mathbb {A}}^1$. Under Panin and Walter’s identification, an element of $\textrm{KQ}^{2,1}({{\,\textrm{Th}\,}}({\mathbb {A}}^1))$ is given by a chain complex of vector bundles *E* together with an isomorphism $E \rightarrow E^\vee [1]$ satisfying certain properties; and the canonical isomorphism $\textrm{KQ}^{0,0}(k) \cong {{\,\textrm{GW}\,}}(k)$ is given by identifying $\langle a\rangle \in {{\,\textrm{GW}\,}}(k)$ with the vector bundle $\mathcal {O}_k$ together with the isomorphism $(\cdot a) :\mathcal {O}_k \rightarrow \mathcal {O}_k$. The Thom isomorphism ${{\,\textrm{th}\,}}$ above is given by multiplication with the Thom class ${{\,\textrm{th}\,}}({\mathbb {A}}^1)$, where the multiplication is induced by taking tensor products. Write ${\mathbb {A}}^1 = {{\,\textrm{Spec}\,}}(k[t])$. Then by [28, Diagram (7.2)], the Thom class ${{\,\textrm{th}\,}}({\mathbb {A}}^1)$ is represented by the Koszul complex

$$\begin{aligned} 0\rightarrow \mathcal {O}_{{\mathbb {A}}^1}\xrightarrow {\cdot t} \mathcal {O}_{{\mathbb {A}}^1}\rightarrow 0 \end{aligned}$$

together with the isomorphism 
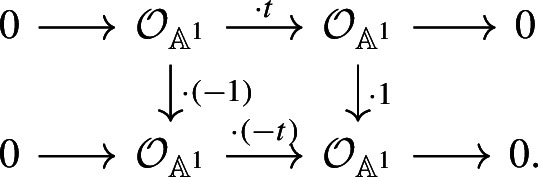


Now, let $a\in k^\times$. There is a canonical isomorphism $({\mathbb {P}}^1,\infty )\cong ({{\,\textrm{Th}\,}}({\mathbb {A}}^1),0)$ in the pointed motivic homotopy category $\mathcal H_\bullet (k)$; see [22, Example 3.1.10]. Under this isomorphism, the map $m_a$ corresponds to the endomorphism $(\cdot a):{{\,\textrm{Th}\,}}({\mathbb {A}}^1) \rightarrow {{\,\textrm{Th}\,}}({\mathbb {A}}^1)$ induced by the ring homomorphism $k[t]\rightarrow k[t], t\mapsto at$. Therefore, the map $(\cdot a)^*:\textrm{KQ}^{2,1}({{\,\textrm{Th}\,}}(\mathbb {A}^1)) \rightarrow \textrm{KQ}^{2,1}({{\,\textrm{Th}\,}}(\mathbb {A}^1))$ sends ${{\,\textrm{th}\,}}({\mathbb {A}}^1)$ to the degree 1 symmetric form 
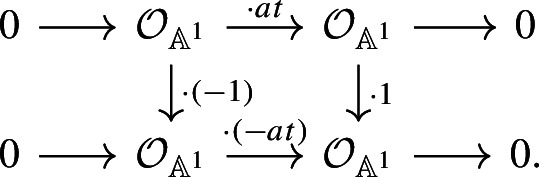
 Furthermore, the Thom isomorphism ${{\,\textrm{th}\,}}$ sends $\langle a\rangle$ to the degree 1 symmetric form 
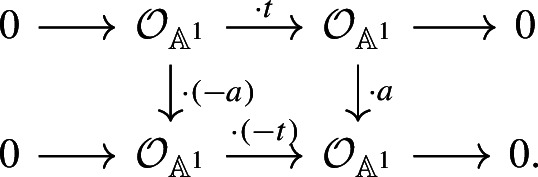
 These symmetric forms are isometric using the morphisms of chain complexes given by 
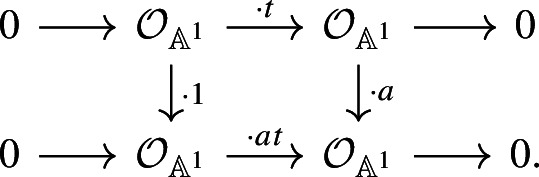
 Hence, we have the following commutative diagram: 
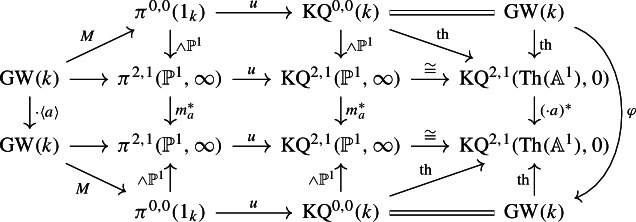


The map $\varphi$ sends $\langle 1\rangle$ to $\langle a \rangle$, by the discussion in the previous paragraph. The triangle 
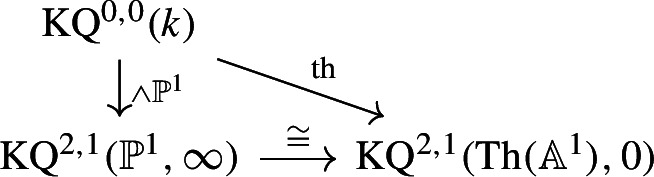
 commutes because the analogous triangle for the ${{\,\textrm{Th}\,}}({\mathbb {A}}^1)$-ring spectrum $\textrm{BO}$ in [28, Section 7, specifically the last diagram on p. 950] commutes by construction and the associated ${\mathbb {P}}^1$-spectrum is $\textrm{KQ}$. We note that this is also the normalisation axiom of SL-oriented cohomology theories, see [1, Definition 3.3 (4)].

Since *u* is a morphism of ring spectra, the composition $u\circ M:{{\,\textrm{GW}\,}}(k)\rightarrow {{\,\textrm{GW}\,}}(k)$ above sends $\langle 1 \rangle$ to $\langle 1 \rangle$. Applying $\varphi$, we get $\langle a \rangle$. On the other hand, starting with $\langle 1 \rangle$ in the ${{\,\textrm{GW}\,}}(k)$ on the left again and multiplying with $\langle a \rangle$, we see that $(u\circ M \circ \cdot \langle a\rangle )(\langle 1 \rangle ) = u\circ M (\langle a \rangle )$. It follows that $u\circ M$ sends $\langle a \rangle$ to $\langle a \rangle$, hence it is the identity, as desired. $\square$

For a discrete valuation ring *A* with residue field *k*, field of fractions *K*, and chosen uniformiser *t*, Morel [23, Lemma 3.16, discussion on p. 57] describes a specialisation homomorphism ${{\,\textrm{sp}\,}}_t:{{\,\textrm{GW}\,}}(K) \rightarrow {{\,\textrm{GW}\,}}(k)$ sending the element $\langle ut^\nu \rangle \in {{\,\textrm{GW}\,}}(K)$ with $u \in A^\times$ and $\nu \in \mathbb {Z}$ to the element $\langle {\bar{u}}\rangle \in {{\,\textrm{GW}\,}}(k)$. Here ${\bar{u}} \in k$ denotes the residue class of *u*.

The following result is probably known in the study of quadratic conductor formulas, but we were unable to find a proof in the literature. We include a proof here for the reader’s convenience.

### Lemma 14

Let *A* be a complete discrete valuation ring with perfect residue field *k* and perfect field of fractions *K*. Choose a uniformiser $t \in A$ of *A*. Let *X* be a smooth, projective scheme over ${{\,\textrm{Spec}\,}}A$ and denote by $X_k$ and $X_K$ its *k*- and *K*-fibre respectively. Then, we have

$$\begin{aligned} \chi (X_k/k) = {{\,\textrm{sp}\,}}_t(\chi (X_K/K)). \end{aligned}$$

Note that this is independent of the choice of uniformiser *t*.

### Proof

We use the six functor formalism on $\mathcal{S}\mathcal{H}(-)$ to prove the lemma, see for example [14]. Let $p_A: X\rightarrow {{\,\textrm{Spec}\,}}(A)$, $p_k: X_k\rightarrow {{\,\textrm{Spec}\,}}(k)$, and $p_K: X_K\rightarrow {{\,\textrm{Spec}\,}}(K)$ be the structure maps of *X* over *A*, $X_k$ over *k*, and $X_K$ over *K*, respectively. Since *X* is smooth and projective, $(p_A)_!(1_{X}) = (p_A)_*(1_{X})$ is strongly dualisable by [14, Theorem 5.22]. Thus, the categorical Euler characteristic of $(p_A)_!(1_{X})$ in $\mathcal{S}\mathcal{H}(A)$ is defined and we denote it by

$$\begin{aligned} \chi ^{\text {cat}}(X):= \chi ^{\text {cat}}((p_A)_!(1_{X})) \in {{\,\textrm{End}\,}}_{\mathcal{S}\mathcal{H}(A)}(1_A) \end{aligned}$$

and similarly $\chi ^{\text {cat}}(X_k):= \chi ^{\text {cat}}((p_k)_!(1_{X_k}))\in {{\,\textrm{End}\,}}_{\mathcal{S}\mathcal{H}(k)}(1_k)$ and $\chi ^{\text {cat}}(X_K):= \chi ^{\text {cat}}((p_K)_!(1_{X_K}))\in {{\,\textrm{End}\,}}_{\mathcal{S}\mathcal{H}(K)}(1_K)$.

Consider the following diagram 
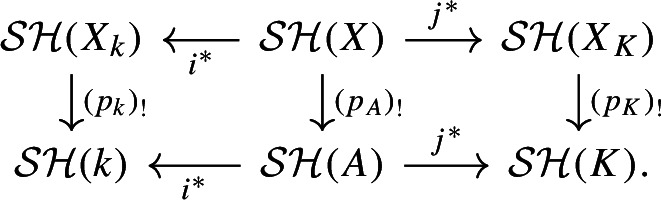
 This diagram is commutative by [14, Theorem 1.1 (3)]. Since $i^*$ and $j^*$ are symmetric monoidal, we get using [16, Remark 2.3 (1)]

$$\begin{aligned} \chi ^{\text {cat}}(X_k)&= \chi ^{\text {cat}}((p_k)_!1_{X_k})\\&=\chi ^{\text {cat}}((p_k)_!i^*1_{X}) \\&= \chi ^{\text {cat}}(i^*(p_A)_!1_{X}) \\&=i^*(\chi ^{\text {cat}}((p_A)_!1_{X})) \\  &= i^*(\chi ^{\text {cat}}(X)) \in {{\,\textrm{End}\,}}_{\mathcal{S}\mathcal{H}(k)}(1_k) \end{aligned}$$

and similarly $$\chi ^{\text {cat}}(X_K) = j^*(\chi ^{\text {cat}}(X))\in {{\,\textrm{End}\,}}_{\mathcal{S}\mathcal{H}(K)}(1_K)$$.

The unit map $u:1_{(-)} \rightarrow \textrm{KQ}_{(-)}$ of Hermitian K-theory induces the map $\pi ^{0,0}u$ on the (0, 0)-th stable homotopy groups. Using this map, we can map each of the above categorical Euler characteristics to the (0, 0)-th stable homotopy group of $\textrm{KQ}_{(-)}$, which is the Grothendieck–Witt ring by for example [12, Introduction]. Abusing notation, let $i^*$ and $j^*$ denote the induced maps on the (0, 0)-th stable homotopy groups of $\textrm{KQ}_{(-)}$. Then, we get $\pi ^{0,0}u\chi ^{\text {cat}}(X_k) = i^*(\pi ^{0,0}u\chi ^{\text {cat}}(X))\in \textrm{KQ}^{0,0}_k = {{\,\textrm{GW}\,}}(k)$ and $\pi ^{0,0}u\chi ^{\text {cat}}(X_K) = j^*(\pi ^{0,0}u\chi ^{\text {cat}}(X))\in KQ^{0,0}_K = {{\,\textrm{GW}\,}}(K)$, where we use Hoyois, Jelisiejew, Nardin, and Yakerson’s result [15, Lemma 7.5] that Hermitian K-theory is stable under base change. The map $\pi ^{0,0}u$ is an isomorphism in $\mathcal{S}\mathcal{H}(k)$ and $\mathcal{S}\mathcal{H}(K)$ by Proposition 13 and the isomorphisms induced by the unit map agree with Morel’s identification of the endomorphisms of the unit in $\mathcal{S}\mathcal{H}(-)$ with the Grothendieck–Witt ring [22, Theorem 6.4.1 and Remark 6.4.2]. Thus, we have $\chi (X_k/k) = \chi _c(X_k/k) = \pi ^{0,0}u\chi ^{\text {cat}}(X_k)$ and $\chi (X_K/K) = \chi _c(X_K/K) = \pi ^{0,0}u\chi ^{\text {cat}}(X_K)$.

We are now in the following situation: 
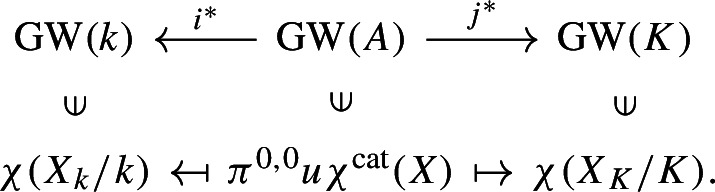


The quadratic form $\pi ^{0,0}u\chi ^{\text {cat}}(X)$ can be expressed as $\sum _i \langle u_i\rangle - \sum _j\langle u'_j\rangle$ for some $u_i, u_j' \in A^\times$ by [30, Chapter 1, Theorem 6.4]. Thus by applying $j^*$, we get $\chi (X_K/K) = \sum _i\langle u_i\rangle - \sum _j\langle u'_j\rangle$, and by applying $i^*$, we get $\chi (X_k/k) = \sum _i\langle {\bar{u}}_i\rangle - \sum _j\langle {\bar{u}}'_j\rangle$. In particular, we get by the definition of the specialisation morphism that $\chi (X_k/k) = {{\,\textrm{sp}\,}}_t(\chi (X_K/K))$. $\square$

### Proposition 15

Assume Theorem 1 holds over all fields of characteristic zero. Then Theorem 1 holds over all fields of characteristic not two.

### Proof

Assume that *k* has positive characteristic. Using [16, Remark 2.1.2], we can assume without loss of generality that *k* is a perfect field. By for example [31, Theorem 3 in Chapter II], we can find a complete discrete valuation ring *A* with residue field *k* and field of fractions *K* of characteristic zero. Now [8, Exposé III, Corollaire 7.4] proves that we can lift *C* to a smooth, projective curve ${\tilde{C}}$ over *A*; that is we get the following diagram of fibre squares: 
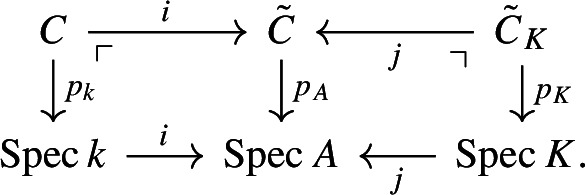
 Since taking ${{\,\textrm{Sym}\,}}^nC$ is compatible with taking the special and the generic fibre, we get the same diagram of fibre squares with ${{\,\textrm{Sym}\,}}^nC$, ${{\,\textrm{Sym}\,}}^n{\tilde{C}}$, and ${{\,\textrm{Sym}\,}}^n{\tilde{C}}_K$ instead of *C*, ${\tilde{C}}$, and ${\tilde{C}}_K$, respectively. Furthermore, ${{\,\textrm{Sym}\,}}^nC, {{\,\textrm{Sym}\,}}^n{\tilde{C}}$ and ${{\,\textrm{Sym}\,}}^n{\tilde{C}}_K$ are smooth and projective by Proposition 11, since ${\tilde{C}}$ is a smooth, projective curve.

By assumption, we know that $\chi ({{\,\textrm{Sym}\,}}^n{\tilde{C}}_K/K)$ is given by the formula in Theorem 1. For any choice of uniformiser $t \in A$, the specialisation map ${{\,\textrm{sp}\,}}_t:{{\,\textrm{GW}\,}}(K) \rightarrow {{\,\textrm{GW}\,}}(k)$ maps this formula to the same formula in ${{\,\textrm{GW}\,}}(k)$. Thus, Lemma 14 yields the claim. $\square$

Proof of Theorem 1 in case n is odd and k has characteristic zero

Since ${{\,\textrm{Sym}\,}}^{n}C$ has odd dimension *n*, its quadratic Euler characteristic is a multiple of the hyperbolic form by the Motivic Gauss–Bonnet Theorem, and thus it is completely determined by its rank. Therefore, Remark 4 implies

$$\begin{aligned} \chi ({{\,\textrm{Sym}\,}}^nC/k) = -\frac{1}{2}\left( {\begin{array}{c}2g-2\\ n\end{array}}\right) \cdot H \end{aligned}$$

as desired. $$\square $$

From now on, assume that $n=2m$ is even and that *k* has characteristic zero. The only reason for the assumption that *k* has characteristic zero is to ensure that *n*! is invertible in *k*. We will compute $\chi ({{\,\textrm{Sym}\,}}^{n}C/k)$ using the Motivic Gauss–Bonnet Theorem. MacDonald [19] has computed the topological Euler characteristic of the symmetric product of a curve over the complex numbers using an explicit basis computation. We follow the same strategy.

### Notation 16

Let $\beta ^\vee \in H^0(C,\mathcal {O}_C)$ be the unit element with respect to the cup product and let $\beta \in H^1(C,\Omega _C^1)$ be the dual element with respect to Serre duality. In particular, the trace map sends $\beta$ to 1. Let $\alpha _1,\dots , \alpha _g$ be a basis for $H^0(C,\Omega _C)$ and let $\alpha _1^\vee , \dots , \alpha _g^\vee$ be the dual basis for $H^1(C,\mathcal {O}_C)$ with respect to Serre duality.

Let $\pi _j:C^n \rightarrow C$ be the projection on the *j*-th component. We write $\alpha _i^{(j)}:= \pi _j^*\alpha _i$ and similarly $\alpha _i^{\vee , (j)}:= \pi _j^*\alpha _i^{\vee }$, $\beta ^{(j)}:= \pi _j^*\beta$, and $\beta ^{\vee , (j)}:= \pi _j^*\beta ^\vee$.

### Remark 17

We have the following multiplication rules for the above generators. Let $i,j\in \{1,\dots , g\}$ be such that $i\ne j$. Then

1. $$\beta \cdot \beta = 0$$
2. $$\beta \cdot \alpha _i = 0$$
3. $$\beta \cdot \beta ^\vee = \beta $$
4. $$\beta \cdot \alpha _i^\vee = 0$$
5. $$\beta ^\vee \cdot \alpha _i = \alpha _i$$
6. $$\beta ^\vee \cdot \beta ^\vee = \beta ^\vee $$
7. $$\beta ^\vee \cdot \alpha ^\vee _i = \alpha ^\vee _i$$
8. $$\alpha _i\cdot \alpha _i = 0$$
9. $$\alpha _i\cdot \alpha _j= 0$$
10. $$\alpha _i^\vee \cdot \alpha _i^\vee = 0$$
11. $$\alpha _i^\vee \cdot \alpha _j^\vee = 0$$
12. $$\alpha _i^\vee \cdot \alpha _i = \beta $$
13. $$\alpha _i^\vee \cdot \alpha _j =0$$

We note that relations 1, 2, 4, 8, 9, 10, and 11 are for degree reasons; relations 3, 5, 6, and 7 are because $\beta ^\vee$ is by definition the unit with respect to the cup product; and relations 12 and 13 hold because the $\alpha _i^\vee$ are by definition the duals of the $\alpha _i$ with respect to Serre duality.

The symmetric group $S_n$ acts on $C^n$ by $\sigma \beta ^{(j)} = \beta ^{(\sigma (j))}$ and $\sigma \alpha _i^{(j)} = \alpha _i^{(\sigma (j))}$ and similarly on the duals for $\sigma \in S_n$, and this extends multiplicatively.

We have ${{\,\textrm{Sym}\,}}^nC = C^n/S_n$. By Proposition 12, we have for $p,q\in \mathbb {Z}_{\ge 0}$ that $H^q({{\,\textrm{Sym}\,}}^nC, \Omega ^p_{{{\,\textrm{Sym}\,}}^nC}) \cong H^q(C^n,\Omega ^p_{C^n})^{S_n}$, where the isomorphism is induced by the quotient map $C^n \rightarrow {{\,\textrm{Sym}\,}}^nC$. In the following, we identify the two *k*-vector spaces.

### Remark 18

We have $H^n(C^n, \Omega _{C^n}^n) \cong k$. A basis is given by the element $\beta ^{(1)} \cdots \beta ^{(n)}$. Therefore, $H^n({{\,\textrm{Sym}\,}}^nC, \Omega _{{{\,\textrm{Sym}\,}}^nC}^n) \cong k$ has basis

$$\begin{aligned}\sum _{\sigma \in S_n} \beta ^{(\sigma (1))} \cdots \beta ^{(\sigma (n))} = n!\beta ^{(1)}\cdots \beta ^{(n)}.\end{aligned}$$

### Lemma 19

Let $0 \le \nu \le m$ and let $I = \{i_1< \dots< i_\nu \},J = \{j_1< \dots < j_\nu \} \subset \{1, \dots , g\}$ be subsets. Define

$$\begin{aligned} a_{IJ}:= \alpha _{i_1}^{(1)} \cdots \alpha _{i_\nu }^{(\nu )}\cdot \alpha _{j_1}^{\vee , (\nu +1)}\cdots \alpha _{j_\nu }^{\vee , (2\nu )}\cdot \beta ^{(2\nu +1)}\cdots \beta ^{(m+\nu )}\cdot \beta ^{\vee , (m+\nu +1)} \cdots \beta ^{\vee , (n)} \end{aligned}$$

in $$H^m(C^n, \Omega _{C^n}^m)$$ and

$$\begin{aligned} \alpha _{IJ}:= \sum _{\sigma \in S_n} \sigma a_{IJ}. \end{aligned}$$

Then, $\alpha _{IJ}$ is an element of $H^m({{\,\textrm{Sym}\,}}^nC, \Omega _{{{\,\textrm{Sym}\,}}^nC}^m)$. Furthermore, $H^m({{\,\textrm{Sym}\,}}^nC, \Omega _{{{\,\textrm{Sym}\,}}^nC}^m)$ has a basis given by $\alpha _{IJ}$, where *I*, *J* runs over all same order subsets $I,J \subset \{1, \dots , g\}$ of order at most *m*.

### Proof

Let $\pi _i: C^n\rightarrow C$ be the projection on the *i*’th coordinate. We have

$$\begin{aligned} \Omega _{C^n} = \pi _1^*\Omega _C\oplus \cdots \oplus \pi _n^*\Omega _C. \end{aligned}$$

This implies

1 $$\begin{aligned} \Omega _{C^n}^m = \Lambda ^m\Omega _{C^n}= \bigoplus _{i_1 + \cdots + i_n = m}\Lambda ^{i_1}\pi _1^*\Omega _C \otimes \cdots \otimes \Lambda ^{i_n}\pi _n^*\Omega _C. \end{aligned}$$

Using (1) and then the Künneth formula, we find

$$\begin{aligned}  &   H^m(C^n,\Omega _{C^n}^m) = \bigoplus _{i_1 + \cdots + i_n = m}H^m(C^n,\Lambda ^{i_1}\pi _1^*\Omega _C \otimes \cdots \otimes \Lambda ^{i_n}\pi _n^*\Omega _C)\\  &   \quad = \bigoplus _{i_1 + \cdots + i_n = m}\bigoplus _{j_1 + \cdots + j_n = m}H^{j_1}(C,\Lambda ^{i_1}\pi _1^*\Omega _C) \otimes \\  &   \qquad \cdots \otimes H^{j_n}(C, \Lambda ^{i_n}\pi _n^*\Omega _C). \end{aligned}$$

We note that $H^{j}(C,\Lambda ^{i}\Omega _C)$ has the basis as in Notation 16

$$\begin{aligned} \left\{ \begin{array}{ll} \beta ^{\vee } & \text { if } j = i = 0,\\ \alpha _1,\dots , \alpha _g & \text { if } j = 0, i = 1,\\ \alpha _1^{\vee },\dots , \alpha _g^{\vee } & \text { if } j = 1, i = 0,\\ \beta & \text { if } j = i = 1. \end{array}\right. \end{aligned}$$

For *I* and *J* same-length tuples $(i_1, \dots , i_\nu )$, $(j_1, \dots j_\nu )$ with $0\le i_1 \le \dots \le i_\nu \le g$ and $0 \le j_1 \le \dots \le j_\nu \le g$ and $\nu \le m$ define

$$\begin{aligned}  &   a_{IJ}:= \alpha _{i_1}^{(1)} \cdots \alpha _{i_\nu }^{(\nu )}\cdot \alpha _{j_1}^{\vee , (\nu +1)}\cdots \alpha _{j_\nu }^{\vee , (2\nu )}\\  &   \quad \cdot \beta ^{(2\nu +1)}\cdots \beta ^{(m+\nu )}\cdot \beta ^{\vee , (m+\nu +1)} \cdots \beta ^{\vee , (n)}. \end{aligned}$$

(The notation $a_{IJ}$ for this is justified as it is a natural extension of the above definition.) Then, we get the basis $(\sigma a_{IJ})_{I,J,\sigma }$ for $H^m(C^n,\Omega _{C^n}^m)$ where *I* and *J* run over same-length tuples of the above form and $\sigma$ runs over the elements of $S_n$ not fixing $a_{IJ}$.

Now, let $I =(i_1, \dots , i_\nu )$ and $J = (j_1, \dots , j_\nu )$ be tuples as above with $\nu \ge 2$. Assume that $i_{\mu } = i_{\mu +1}$ for some $\mu$. Then, we note that $\sigma a_{IJ} = -a_{IJ}$ for $\sigma$ the transposition swapping $\mu$ and $\mu + 1$.

Thus, if $v \in H^m({{\,\textrm{Sym}\,}}^nC, \Omega ^m_{{{\,\textrm{Sym}\,}}^nC}) = H^m(C^n, \Omega ^m_{C^n})^{S_n}$, the $a_{IJ}$-coefficient of *v* vanishes for such *I*, *J*. Analogously, we get the same vanishing if $j_\mu = j_{\mu +1}$.

In order to construct a basis of $H^m({{\,\textrm{Sym}\,}}^nC, \Omega ^m_{{{\,\textrm{Sym}\,}}^nC})$, we can therefore restrict to the $a_{IJ}$ where $i_1< \dots < i_\nu$ and $j_1< \dots < j_\nu$. Since, $S_n$ acts freely on these elements, we take sums under all $\sigma \in S_n$, to construct a basis of the $S_n$ fixed-points. This is the desired basis. $\square$

### Lemma 20

Let $\sigma \in S_n$ and $I,J, I', J' \subset \{1, \dots , g\}$, with $\nu = |I| = |J| \le m$ and $|I'| = |J'| \le m$. In the notation of Lemma 19, we have

$$\begin{aligned} a_{IJ} \cdot \sigma a_{I'J'} = (-1)^\nu \beta ^{(1)}\cdots \beta ^{(n)} \in H^n(C^n, \Omega _{C^n}^n) \end{aligned}$$

whenever $I = J', J = I'$ and $\sigma$ satisfies

- $\sigma (i) = \nu +i$ and $\sigma (\nu +i) = i$ for $1 \le i \le \nu$ and
- $$\sigma (\{2\nu +1, \dots , m+ \nu \}) = \{m+ \nu + 1, \dots , n\}$$.

Otherwise $$a_{IJ} \cdot \sigma a_{I'J'} = 0$$.

### Proof

The last assertion follows from the multiplication table in Remark 17.

Assume that $I = J', J = I'$ and that $\sigma$ satisfies the condition of the lemma. Then, we have

$$\begin{aligned} \sigma a_{JI}= &   \alpha _{j_1}^{(\nu + 1)} \cdots \alpha _{j_\nu }^{(2\nu )}\cdot \alpha _{i_1}^{\vee , (1)}\cdots \alpha _{i_\nu }^{\vee , (\nu )}\\  &   \cdot \beta ^{(\sigma (2\nu +1))}\cdots \beta ^{(\sigma (m+\nu ))}\cdot \beta ^{\vee , (\sigma (m+\nu +1))} \cdots \beta ^{\vee , (\sigma (n))}. \end{aligned}$$

Since the degrees of $\beta ^{(i)}$ and $\beta ^{\vee ,(i)}$ are even, we can rearrange these without introducing a sign. Thus, the above equation can be rewritten as

$$\begin{aligned}  &   \sigma a_{JI} = \alpha _{j_1}^{(\nu + 1)} \cdots \alpha _{j_\nu }^{(2\nu )}\cdot \alpha _{i_1}^{\vee , (1)}\cdots \alpha _{i_\nu }^{\vee , (\nu )}\cdot \\  &   \quad \beta ^{\vee , (2\nu +1)}\cdots \beta ^{\vee , (m+\nu )}\cdot \beta ^{(m+\nu +1)} \cdots \beta ^{(n)}. \end{aligned}$$

Define $B':= \beta ^{\vee , (2\nu +1)}\cdots \beta ^{\vee , (m+\nu )}\cdot \beta ^{(m+\nu +1)} \cdots \beta ^{(n)}$ and, similarly, define $B:= \beta ^{(2\nu +1)}\cdots \beta ^{(m+\nu )}\cdot \beta ^{\vee , (m+\nu +1)} \cdots \beta ^{\vee , (n)}$. Then, since *B* and $B'$ have even degree, we have that

$$\begin{aligned} a_{IJ} \cdot \sigma a_{JI}&= \alpha _{i_1}^{(1)} \cdots \alpha _{i_\nu }^{(\nu )}\cdot \alpha _{j_1}^{\vee , (\nu +1)}\cdots \alpha _{j_\nu }^{\vee , (2\nu )} \cdot B \cdot \alpha _{j_1}^{(\nu + 1)} \cdots \alpha _{j_\nu }^{(2\nu )}\cdot \alpha _{i_1}^{\vee , (1)}\cdots \alpha _{i_\nu }^{\vee , (\nu )}\cdot B'\\&= \alpha _{i_1}^{(1)} \cdots \alpha _{i_\nu }^{(\nu )}\cdot \alpha _{j_1}^{\vee , (\nu +1)}\cdots \alpha _{j_\nu }^{\vee , (2\nu )} \cdot \alpha _{j_1}^{(\nu + 1)} \cdots \alpha _{j_\nu }^{(2\nu )}\cdot \alpha _{i_1}^{\vee , (1)}\cdots \alpha _{i_\nu }^{\vee , (\nu )}\cdot B\cdot B' \end{aligned}$$

All $\alpha ^{(i)}$’s and $\alpha ^{\vee , (i)}$’s have odd degree. Thus, if we rearrange these elements by a permutation $\tau$, we have to multiply with $(-1)^{{{\,\textrm{sgn}\,}}(\tau )}$. Therefore, if we rearrange

$$\begin{aligned} \alpha _{i_1}^{(1)} \cdots \alpha _{i_\nu }^{(\nu )}\cdot \alpha _{j_1}^{\vee , (\nu +1)}\cdots \alpha _{j_\nu }^{\vee , (2\nu )} \cdot \alpha _{j_1}^{(\nu + 1)} \cdots \alpha _{j_\nu }^{(2\nu )}\cdot \alpha _{i_1}^{\vee , (1)}\cdots \alpha _{i_\nu }^{\vee , (\nu )}\cdot B\cdot B' \end{aligned}$$

as

$$\begin{aligned} \alpha _{i_1}^{(1)} \cdots \alpha _{i_\nu }^{(\nu )}\cdot \alpha _{j_1}^{\vee , (\nu +1)}\cdots \alpha _{j_\nu }^{\vee , (2\nu )} \cdot \alpha _{i_1}^{\vee , (1)}\cdots \alpha _{i_\nu }^{\vee , (\nu )}\cdot \alpha _{j_1}^{(\nu + 1)} \cdots \alpha _{j_\nu }^{(2\nu )}\cdot B\cdot B', \end{aligned}$$

we are swapping $\nu$ times, so this introduces the sign $(-1)^\nu$. If we next rearrange this into

$$\begin{aligned} \alpha _{i_1}^{(1)}\alpha ^{\vee ,(1)}_{i_1} \cdots \alpha _{i_\nu }^{(\nu )}\alpha _{i_\nu }^{\vee , (\nu )}\cdot \alpha _{j_1}^{\vee , (\nu +1)}\alpha ^{(\nu +1)}_{j_1}\cdots \alpha _{j_\nu }^{\vee , (2\nu )}\alpha _{j_\nu }^{(2\nu )}\cdot B\cdot B', \end{aligned}$$

we are moving $\alpha _{i_1}^{\vee , (1)}$ by $2\nu -1$ elements to the left, then we are moving $\alpha _{i_2}^{\vee , (2)}$ by $2\nu -2$ elements to the left, $\alpha _{i_3}^{\vee , (3)}$ by $2\nu -3$ elements, and so on. Therefore, the resulting permutation has sign $(-1)^\eta$ where

$$\begin{aligned} \eta = \sum _{i=1}^{2\nu } 2\nu -i = \sum _{i=0}^{2\nu -1} i = \frac{2\nu (2\nu -1)}{2} = \nu (2\nu -1). \end{aligned}$$

Thus, we have

$$\begin{aligned} a_{IJ} \cdot \sigma a_{JI}&= \underset{=1}{\underbrace{(-1)^{\nu }(-1)^{\nu (2\nu -1)}}}\cdot \alpha _{i_1}^{(1)}\alpha ^{\vee ,(1)}_{i_1} \cdots \alpha _{i_\nu }^{(\nu )}\alpha _{i_\nu }^{\vee , (\nu )}\\&\cdot \alpha _{j_1}^{\vee , (\nu +1)}\alpha ^{(\nu +1)}_{j_1}\cdots \alpha _{j_\nu }^{\vee , (2\nu )}\alpha _{j_\nu }^{(2\nu )}\cdot B\cdot B'\\&= \underset{=\beta ^{(1)}}{\underbrace{\alpha _{i_1}^{(1)}\alpha ^{\vee ,(1)}_{i_1}}} \cdots \underset{=\beta ^{(\nu )}}{\underbrace{\alpha _{i_\nu }^{(\nu )}\alpha _{i_\nu }^{\vee , (\nu )}}}\cdot \underset{=-\beta ^{(\nu +1)}}{\underbrace{\alpha _{j_1}^{\vee , (\nu +1)}\alpha ^{(\nu +1)}_{j_1}}}\cdots \underset{=-\beta ^{(2\nu )}}{\underbrace{\alpha _{j_\nu }^{\vee , (2\nu )}\alpha _{j_\nu }^{(2\nu )}}}\cdot B\cdot B'\\&= (-1)^\nu \beta ^{(1)}\cdots \beta ^{(2\nu )} \underset{= \beta ^{(2\nu +1)}\cdots \beta ^{(n)}}{\underbrace{B\cdot B'}}\\&= (-1)^\nu \beta ^{(1)}\cdots \beta ^{(n)}. \qquad \qquad \hspace{5cm} \end{aligned}$$

$$\square $$

### Lemma 21

Let $f:X \rightarrow Y$ be a finite surjective morphism of degree *d* between smooth, projective, *n*-dimensional schemes *X* and *Y* over *k*. Then, we have the commutative diagram 
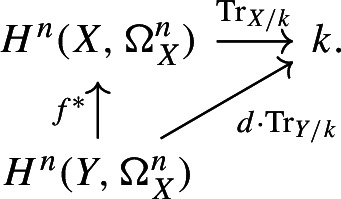


### Proof

This argument was explained to the first named author by Marc Levine. Let $f_*: H^n(X,\Omega ^n_X)\rightarrow H^n(Y,\Omega ^n_Y)$ be the pushforward as constructed by Srinivas [32]. We note that by [32, Theorem 1 (e)], for a smooth, proper scheme *Z* of dimension *d* over *k*, the trace map $\text {Tr}_{Z/k}: H^d(Z,\Omega _Z^d)\rightarrow k$ is equal to $p_*$ where $p:Z\rightarrow {{\,\textrm{Spec}\,}}(k)$ is the structure map. Therefore, by [32, Theorem 1 (b)], the diagram 
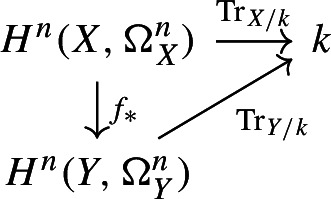
 commutes. Furthermore, by [32, Theorem 1 (d) and (f)], we have $f_*f^*= d\cdot \operatorname {id}$. This yields the desired result. $\square$

Proof of Theorem 1*in case n=2 m is even and k has characteristic zero* We will apply the Motivic Gauss–Bonnet Theorem, and thus, we need to compute the form 
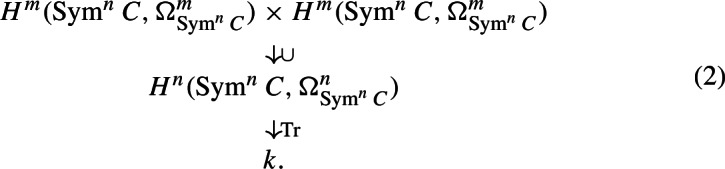
 By Lemma 19, the vector space $H^m({{\,\textrm{Sym}\,}}^nC, \Omega _{{{\,\textrm{Sym}\,}}^nC}^m)$ has basis $\alpha _{IJ}$ for *I*, *J* running over the same size subsets $I,J \subset \{1, \dots , g\}$ with $|I| = |J| \le m$. By Lemma 20, we know that $\alpha _{IJ}\alpha _{I'J'} = 0$ whenever $I \ne J'$ or $J \ne I'$. Thus, it remains to compute the cup product $\alpha _{IJ}\alpha _{JI}$. For this, note that

$$\begin{aligned} \alpha _{IJ}\alpha _{JI} = \sum _{\sigma ,\tau \in S_n} (\sigma a_{IJ})\cdot (\tau a_{JI})= \sum _{\sigma , \tau \in S_n} \sigma (a_{IJ}\cdot (\sigma ^{-1}\tau )a_{JI}) = \sum _{\sigma , \tau \in S_n} \sigma (a_{IJ}\cdot \tau a_{JI}) \end{aligned}$$

where in the last step, we replace $\sigma ^{-1}\tau$ by $\tau$. Since $a_{IJ}\cdot \tau a_{JI}$ is a multiple of $\beta ^{(1)}\cdots \beta ^{(n)}$ and $S_n$ acts trivially on this element, we get by Lemma 20

$$\begin{aligned} \alpha _{IJ}\alpha _{JI}&= \sum _{\sigma , \tau \in S_n} \sigma (a_{IJ}\cdot \tau a_{JI})\\&= n!\cdot \sum _{\tau \in S_n} a_{IJ}\cdot \tau a_{JI} \\&= n!\cdot \sum _{\tau \in S_n'} (-1)^{|I|}\beta ^{(1)}\cdots \beta ^{(n)} \\&= (-1)^{|I|}\cdot n!\cdot |S_n'|\cdot \beta ^{(1)}\cdots \beta ^{(n)} \end{aligned}$$

where $S_n' \subset S_n$ is the subset of all permutations satisfying the non-vanishing condition of Lemma 20 for *I* and *J*. Thus, in order to compute the product, we have to determine the cardinality of $S_n'$. Note that

$$\begin{aligned} S_n' = \{\tau \sigma \mid \sigma \in S_{m-|I|}\times S_{m-|I|}\}, \end{aligned}$$

where we embed $S_{m-|I|}\times S_{m-|I}$ in $S_n$ by letting it permute the last $2(m-|I|)$ elements of $\{1, \dots , n\}$ and where $\tau \in S_n$ is the permutation defined as follows: We set $\tau (i) = |I|+i$ and $\tau (|I|+i) = i$ for $1 \le i \le |I|$, and $\tau (2|I|+j) = m+ |I|+ j$ and $\tau (m+|I|+j) = 2|I| + j$ for $1 \le j \le m-|I|$. Thus, $|S_n'| = |S_{m-|I|}\times S_{m-|I|}| = ((m-|I|)!)^2$, and we have

$$\begin{aligned} \alpha _{IJ}\alpha _{JI} = (-1)^{|I|}\cdot n!\cdot ((m-|I|)!)^2\cdot \beta ^{(1)}\cdots \beta ^{(n)}. \end{aligned}$$

Applying Lemma 21 to the quotient map $C^n\rightarrow {{\,\textrm{Sym}\,}}^nC$ yields ${{\,\textrm{Tr}\,}}(\beta ^{(1)}\cdots \beta ^{(n)}) = (n!)^{-1}$. We obtain

$$\begin{aligned} {{\,\textrm{Tr}\,}}(\alpha _{IJ}\alpha _{I'J'}) = \left\{ \begin{array}{cc} (-1)^{|I|}\cdot ( (m-|I|)!)^2 &  \text {if }I=J' \text { and } J = I'\\ 0 &  \text {otherwise.} \end{array}\right. \end{aligned}$$

Thus, the matrix representing the bilinear form with respect to the basis $(\alpha _{IJ})$ takes the form of a diagonal block matrix with blocks $A_{\{I,J\}}$ for $I,J \subset \{1, \dots , g\}$ same-size subsets of order at most *m*. Each block $A_{\{I,J\}}$ takes the form

$$\begin{aligned} A_{\{I,J\}} = \begin{pmatrix} (-1)^{|I|}\cdot ((m-|I|)!)^2 \end{pmatrix} \end{aligned}$$

if $$I = J$$ and

$$\begin{aligned} A_{\{I,J\}} = \begin{pmatrix} 0 &  (-1)^{|I|}\cdot ( (m-|I|)!)^2\\ (-1)^{|I|}\cdot ( (m-|I|)!)^2 &  0 \end{pmatrix} \end{aligned}$$

if $I\ne J$. The matrix $A_{\{I,I\}}$ represents the form $\langle (-1)^{|I|}\cdot ( (m-|I|)!)^2\rangle = \langle (-1)^{|I|}\rangle \in {{\,\textrm{GW}\,}}(k)$, and the matrix $A_{\{I,J\}}$ with $I \ne J$ represents the hyperbolic form in ${{\,\textrm{GW}\,}}(k)$. Thus, we have that the quadratic form *q* in (2) is given by

$$\begin{aligned} q=\sum _{I \subset \{1, \dots , g\}} \langle (-1)^{|I|}\rangle + l\cdot H \end{aligned}$$

for some $l \in \mathbb {Z}$ where the sum only runs over subsets of order at most *m*. Since $\{1, \dots , g\}$ contains $$\left( {\begin{array}{c}g\\ i\end{array}}\right) $$ subsets of order *i*, we get

$$\begin{aligned} q = \sum _{i=0}^m\left( {\begin{array}{c}g\\ i\end{array}}\right) \langle (-1)^i\rangle + lH. \end{aligned}$$

Combining this computation with Remark 4 yields the first formula of the theorem. For the second formula, we plug in $H = \langle 1\rangle + \langle -1\rangle$ and then note that

$$\begin{aligned} \sum _{i=0}^m\left( {\begin{array}{c}g\\ i\end{array}}\right) (-1)^i = (-1)^m\left( {\begin{array}{c}g-1\\ m\end{array}}\right) . \hspace{5cm} \end{aligned}$$

$$\square $$

## Compatibility with the power structure

Recall that a power structure on a commutative ring *R* is a map $\phi : (1+tR[[t]])\times R\rightarrow 1+tR[[t]]$, sometimes written as $\phi (f(t),r) = f(t)^r$, satisfying certain axioms, see for example [26, Section 2] for a precise definition. We only consider finitely determined power structures in the sense of [26]. In this setting, it suffices to define $(1-t)^{-r}:= \sum _{n=0}^\infty a_n(r)t^n$ for $r \in R$, see [10, Proposition 1]. That is, by [26, Corollary 2.5] a power structure can be given by functions $a_n: R\rightarrow R$ for $n\in {\mathbb {Z}}_{\ge 0}$ such that:

- $$a_0 = 1$$,
- $a_1$ is the identity,
- $$a_n(0) = 0$$ for all $$n\ge 1$$,
- $a_n(1) = 1$ for all $n\ge 1$, and
- $$a_n(r+s) = \sum _{i=0}^na_i(r)a_{n-i}(s)$$ for all $$r,s\in R$$.

A ring homomorphism $\phi : R\rightarrow R'$ between rings that both carry a power structure is said to respect the power structures if $\phi (a_n^R(r)) = a_n^{R'}(\phi (r))$ for all $r\in R$ and $n\in {\mathbb {Z}}_{\ge 0}$.

Let $K_0(\text {Var}_k)^{rs}$ be $K_0(\text {Var}_k)$ with radicial surjections inverted, see [24, Appendix A.3]. We note that $K_0(\text {Var}_k)^{rs}=K_0(\text {Var}_k)$ if *k* has characteristic zero by [24, Proposition A.24]. There is a power structure on $K_0(\text {Var}_k)^{rs}$ given by $a_n([X]) = [{{\,\textrm{Sym}\,}}^nX]$, which can be checked using [24, Lemma 7.29]. We note that this was first proven by Gusein–Zade et al. [9] in characteristic zero, using the standard stratification of the symmetric power to define the power structure. If *k* has positive characteristic, this stratification is only isomorphic to the symmetric power after inverting radicial surjective morphisms, which is why we consider $K_0(\text {Var}_k)^{rs}$ instead of $K_0(\text {Var}_k)$.

By work of Bejleri and McKean [3, Corollary 5.4], the compactly supported ${\mathbb {A}}^1$-Euler characteristic factors through $K_0(\text {Var}_k)^{rs}$.

Pajwani and Pál [26] constructed a power structure on ${{\,\textrm{GW}\,}}(k)$ given by

$$\begin{aligned} a_n(\langle \alpha \rangle ) = \langle \alpha ^n\rangle + \frac{n(n-1)}{2}(\langle \alpha \rangle + \langle 2 \rangle - \langle 1 \rangle - \langle 2\alpha \rangle ). \end{aligned}$$

### Remark 22

This power structure agrees with McGarraghy’s non-factorial power structure [20] on ${{\,\textrm{GW}\,}}(k)$ on the subgroup generated by all $\langle \alpha \rangle$ satisfying $a_n(\langle \alpha \rangle ) = \langle \alpha ^n\rangle$, see [26, Lemma 2.9, Lemma 3.27 and Corollary 3.28]. The quadratic forms $\langle 1 \rangle$ and $\langle -1 \rangle$ are in this subgroup.

Pajwani and Pál prove that $\chi _c: K_0(\text {Var}_k)^{rs}\rightarrow {{\,\textrm{GW}\,}}(k)$ respects the power structures for zero dimensional schemes, i.e. $\chi _c({{\,\textrm{Sym}\,}}^nX/k) = a_n(\chi _c(X/k))$ for *X* a zero dimensional scheme. This was extended to a larger class of schemes by Pajwani, Rohrbach, and the second named author in [27], who also showed compatibility for curves of genus 0 and 1. We now extend this result to all curves, i.e. we check that all curves are symmetrizable in the sense of [27, Definition 4.1]. We note that in [26, 27], it is assumed that the base field is of characteristic zero, but by the results of Bejleri-McKean, this assumption can be weakened to fields that are not of characteristic 2.

For *C* a smooth, projective curve of genus *g*, we have $\chi (C/k) = (1-g)H$ (see Remark 2). We therefore need to evaluate $a_n$ on negative multiples of *H*. Note that McGarraghy [20, Corollary 4.13 and 4.14] gave a complete description of what happens for positive multiples.

### Lemma 23

Let $$l\in {\mathbb {Z}}_{\ge 1}$$. Then $$a_n(-lH) = (-1)^n \sum _{i=0}^n\left( {\begin{array}{c}l\\ i\end{array}}\right) \left( {\begin{array}{c}l\\ n-i\end{array}}\right) \langle -1 \rangle ^{n-i}$$.

### Proof

One can prove by induction on *l* that

$$\begin{aligned} a_n(-l\langle 1 \rangle ) = (-1)^n\left( {\begin{array}{c}l\\ n\end{array}}\right) \langle 1 \rangle \text { and } a_n(-l\langle -1 \rangle ) = (-1)^n\left( {\begin{array}{c}l\\ n\end{array}}\right) \langle -1 \rangle ^n. \end{aligned}$$

We obtain

$$\begin{aligned} a_n(-lH)&= \sum _{i=0}^na_i(-l\langle 1 \rangle )a_{n-i}(-l\langle -1 \rangle ) \\&= (-1)^n\sum _{i=0}^n\left( {\begin{array}{c}l\\ i\end{array}}\right) \left( {\begin{array}{c}l\\ n-i\end{array}}\right) \langle -1\rangle ^{n-i} \end{aligned}$$

as desired. $$\square $$

### Remark 24

We note that for $l,n\in {\mathbb {Z}}_{\ge 1}$, the rank of $a_n(-lH)$ equals

$$\begin{aligned} (-1)^n\sum _{i=0}^n\left( {\begin{array}{c}l\\ i\end{array}}\right) \left( {\begin{array}{c}l\\ n-i\end{array}}\right) = (-1)^n\left( {\begin{array}{c}2l\\ n\end{array}}\right) \end{aligned}$$

by the Chu-Vandermonde identity. Let *C* be a smooth, projective curve over *k* of genus *g*, then we know from Theorem 1 that $$\text {rank}(\chi ({{\,\textrm{Sym}\,}}^nC/k)) = (-1)^n\left( {\begin{array}{c}2\,g-2\\ n\end{array}}\right) $$. Plugging in $l = g-1$ to the above formula, we find that $$\text {rank}(a_n(1-g)H) = (-1)^n\left( {\begin{array}{c}2g-2\\ n\end{array}}\right) $$. Therefore, we see that the ranks of $\chi ({{\,\textrm{Sym}\,}}^nC/k)$ and $a_n(\chi (C/k))$ agree.

There is another way to compute $a_n(-lH)$, suggested by Marc Levine.

### Lemma 25

Let $l\in {\mathbb {Z}}_{\ge 1}$. Then $a_n(-lH)$ is the coefficient of $t^n$ in the polynomial $(\langle 1 \rangle -Ht+\langle -1 \rangle t^2)^l$.

### Proof

For $\langle a \rangle \in {{\,\textrm{GW}\,}}(k)$, let $a_t(\langle a \rangle ):= (\langle 1 \rangle -\langle 1\rangle t)^{-\langle a \rangle }:= \sum _{i=0}^\infty a_i(\langle a \rangle )t^i$. Then $a_t(\langle a \rangle + \langle b \rangle ) = a_t(\langle a\rangle )a_t(\langle b\rangle )$. As $a_t(\langle 1 \rangle ) = \sum _{i=0}^\infty \langle 1 \rangle t^i$, we see from

$$\begin{aligned} \langle 1 \rangle = a_t(\langle 1\rangle - \langle 1 \rangle ) = a_t(\langle 1 \rangle )a_t(-\langle 1 \rangle ) \end{aligned}$$

that $a_t(-\langle 1 \rangle ) = \langle 1 \rangle -\langle 1 \rangle t$. One can similarly show that $a_t(-\langle -1 \rangle ) = \langle 1 \rangle -\langle -1 \rangle t.$ This yields

$$\begin{aligned} a_t(-lH)&= ((\langle 1 \rangle -\langle 1 \rangle t)(\langle 1 \rangle -\langle -1\rangle t))^l \\&= (\langle 1 \rangle -Ht+\langle -1 \rangle t^2)^l \end{aligned}$$

as desired. $$\square $$

### Proposition 26

Let *C* be a smooth, projective curve of genus *g* and let $n\in {\mathbb {Z}}_{\ge 0}$. Then

$$\begin{aligned} \chi ({{\,\textrm{Sym}\,}}^nC/k)=a_n(\chi (C/k)). \end{aligned}$$

### Proof

We note that if $g=0$, then $C\cong {\mathbb {P}}^1$ in which case the statement holds, either by direct calculation or by [27, Theorem 1.1]. If $g=1$ then $\chi (C/k) = 0$, so $a_n(\chi (C/k))=0$ for all $n\ge 1$, and by Theorem 1, the same holds for $\chi ({{\,\textrm{Sym}\,}}^nC/k)$. One can alternatively use [27, Theorem 1.2]. We therefore assume that $g\ge 2$ from now on.

The two forms have the same rank by Remark 24. Suppose that *n* is odd. Then $\chi ({{\,\textrm{Sym}\,}}^nC/k)$ is hyperbolic by the Motivic Gauss–Bonnet Theorem, see Theorem 9. Furthermore, $a_n(\chi (C/k))$ is hyperbolic by [27, Lemma 2.10]. This yields the result in the case that *n* is odd. Thus, assume that $n = 2\,m$ is even from now on.

Note that both forms only consist of terms $\langle 1 \rangle$ and $\langle -1 \rangle$. For $\chi ({{\,\textrm{Sym}\,}}^nC/k)$, this follows from Theorem 1, and for $a_n(\chi (C/k))$, we see this from Lemma 23.

Therefore, given that we know the ranks are the same, the two are the same if for both forms the difference between the number of $\langle 1\rangle$’s and $\langle -1\rangle$’s is the same. Note that if we work over the real numbers this difference is the signature of the quadratic form. By Theorem 1, this difference is $$(-1)^m\left( {\begin{array}{c}g-1\\ m\end{array}}\right) $$ for $\chi ({{\,\textrm{Sym}\,}}^{2\,m}C/k)$.

On the other hand, denote this difference for $a_n((1-g)H)$ by *s*. Lemma 25 yields that $a_n((1-g)H)$ is the coefficient of $t^n$ in

$$\begin{aligned} (\langle 1 \rangle -Ht+\langle -1 \rangle t^2)^{g-1}&= \sum _{i=0}^{g-1} \left( {\begin{array}{c}g-1\\ i\end{array}}\right) (\langle 1 \rangle + \langle -1 \rangle t^2)^i (-Ht)^{g-1-i}. \end{aligned}$$

All summands with $i\ne g-1$ are a multiple of *H* and thus do not contribute to *s*. Thus, *s* is completely determined by the degree 2*m*-term of

$$\begin{aligned} (\langle 1\rangle + \langle -1\rangle t^2)^{g-1}&= \sum _{i=0}^{g-1} \left( {\begin{array}{c}g-1\\ i\end{array}}\right) \langle -1 \rangle ^{i}t^{2i}, \end{aligned}$$

which is $$\left( {\begin{array}{c}g-1\\ m\end{array}}\right) \langle -1\rangle ^mt^{2m}$$. This shows $$s = (-1)^m\left( {\begin{array}{c}g-1\\ m\end{array}}\right) $$, which proves the proposition. $\square$

We can extend this to show that the power structure is respected for all curves.

### Theorem 27

Let *C* be a curve and let $n\in {\mathbb {Z}}_{\ge 0}$. Then

$$\begin{aligned} \chi _c({{\,\textrm{Sym}\,}}^nC/k) = a_n(\chi _c(C/k)). \end{aligned}$$

### Proof

We can assume without loss of generality that *k* is perfect, see Remark 10, which implies that *C* is generically smooth. Let $\tilde{C}$ be the normalization of *C*. Let $S\subset C$ be the locus of singular points on *C* and let $\tilde{S}\subset \tilde{C}$ be the points lying over *S*. Then, the normalization map $\pi : \tilde{C}\rightarrow C$ is an isomorphism outside of the singular points of *C* and so

$$\begin{aligned} [C] = [\tilde{C}] - [\tilde{S}] + [S] \end{aligned}$$

in $K_0(\text {Var}_k)^{rs}$. By Proposition 26, the power structure is respected on $\tilde{C}$. Also, as *S* and $\tilde{S}$ are zero dimensional, the power structure is respected on those by [26, Theorem 4.1]. By [26, Lemma 2.9], the set of elements of $K_0(\text {Var}_k)^{rs}$ for which the power structure is respected forms a subgroup, implying that the power structure is respected for *C*. $\square$

## Declarations

### Data sharing

Data sharing not applicable to this article as no data sets were generated or analyzed during the current study.
